# Metabolic Acidosis in CKD: Pathogenesis, Adverse Effects, and Treatment Effects

**DOI:** 10.3390/ijms25105187

**Published:** 2024-05-10

**Authors:** Kalani L. Raphael

**Affiliations:** 1 Division of Nephrology & Hypertension, Department of Internal Medicine, University of Utah, Salt Lake City, UT 84112, USA; kalani.raphael@hsc.utah.edu; 2 Medicine Section, Veterans Affairs Salt Lake City Health Care System, Salt Lake City, UT 84148, USA

**Keywords:** metabolic acidosis, subclinical metabolic acidosis, chronic kidney disease, acid–base balance, sodium bicarbonate, diet, nutrition, end-stage kidney disease, tubulointerstitial fibrosis

## Abstract

Metabolic acidosis is a frequent complication of chronic kidney disease and is associated with a number of adverse outcomes, including worsening kidney function, poor musculoskeletal health, cardiovascular events, and death. Mechanisms that prevent metabolic acidosis detrimentally promote further kidney damage, creating a cycle between acid accumulation and acid-mediated kidney injury. Disrupting this cycle through the provision of alkali, most commonly using sodium bicarbonate, is hypothesized to preserve kidney function while also mitigating adverse effects of excess acid on bone and muscle. However, results from clinical trials have been conflicting. There is also significant interest to determine whether sodium bicarbonate might improve patient outcomes for those who do not have overt metabolic acidosis. Such individuals are hypothesized to be experiencing acid-mediated organ damage despite having a normal serum bicarbonate concentration, a state often referred to as subclinical metabolic acidosis. Results from small- to medium-sized trials in individuals with subclinical metabolic acidosis have also been inconclusive. Well-powered clinical trials to determine the efficacy and safety of sodium bicarbonate are necessary to determine if this intervention improves patient outcomes.

## 1. Introduction

Systemic pH is tightly regulated to maintain normal cellular function. Kidneys and lungs are the principal organs responsible for keeping arterial pH in the range of 7.40. CO_2_ retention through insufficient respiratory ventilation leads to respiratory acidosis, while the accumulation of excess non-volatile acid leads to metabolic acidosis. Acute metabolic acidosis can be seen in a variety of settings, including diarrhea, toxic alcohol ingestions, lactic acidosis, and ketoacidosis. The most common cause of chronic metabolic acidosis, the focus of this review, is chronic kidney disease (CKD), which affects nearly 900 million individuals worldwide [1]. Chronic metabolic acidosis in CKD is associated with excess risks of, among others, CKD progression, cardiovascular disease (CVD), and musculoskeletal complications [2,3,4,5,6,7,8,9,10,11,12,13,14,15]. Roughly 15% of those with CKD have chronic metabolic acidosis, amounting to 135 million individuals worldwide who are at increased risk of these complications [16,17]. The treatment of chronic metabolic acidosis in CKD with alkali therapy is straightforward and generally safe, but its effectiveness still remains unclear.

## 2. Genesis of Chronic Metabolic Acidosis in CKD

**Figure 1:**
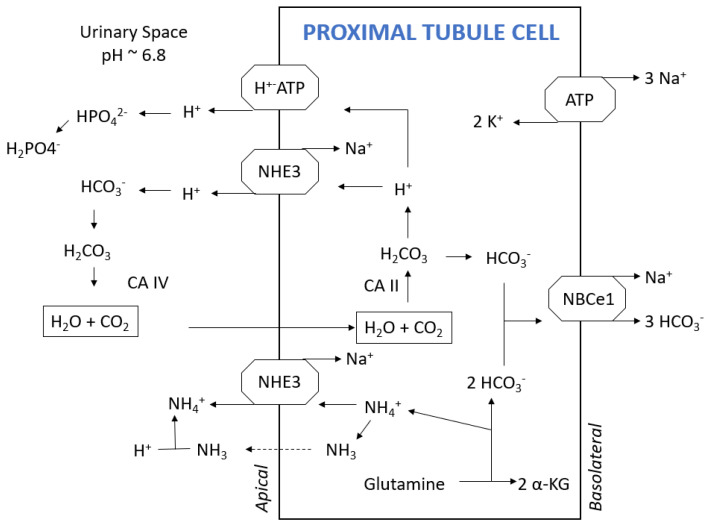
Key components of acid–base regulation in a proximal tubule cell (PTC). Monohydrogen phosphate (HPO_4_^2−^) and bicarbonate (HCO_3_^−^) are filtered and bind to protons (H^+^) secreted by the H^+^-ATPase or the sodium–hydrogen antiporter (NHE3) on the luminal side of the PTC, forming dihydrogen phosphate (H_2_PO_4_^−^) and carbonic acid (H_2_CO_3_), respectively. Carbonic acid is converted to H_2_O and CO_2_ by carbonic anhydrase (CA) IV, which then move intracellularly where CA II re-forms carbonic acid. Carbonic acid dissociates to a proton and bicarbonate, where the proton can be reused by the H^+^-ATPase or NHE3. Glutamine is metabolized to two ammonium (NH_4_^+^) and two α-ketoglutarate (α-KG) molecules, forming two bicarbonate anions in the process. Three bicarbonate anions are co-transported with sodium on the sodium-bicarbonate co-transporter (NBCe1) on the basolateral side. Ammonium exits the PTC as a substitute for protons on NHE3 or as ammonia (NH_3_) by diffusion across the cell membrane where it can bind to a proton, forming ammonium.

**Figure 2:**
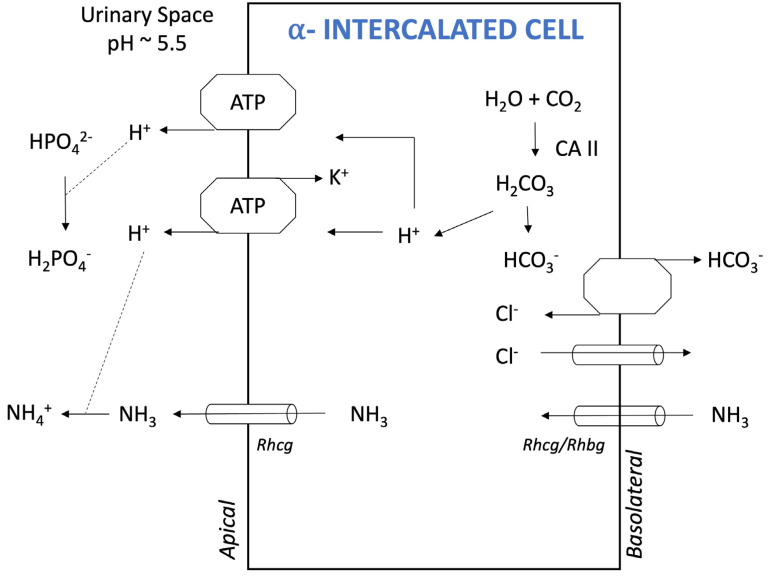
Key components of acid–base regulation in an α-intercalated cell (IC). Under most circumstances, all the bicarbonate (HCO_3_^−^) that has been filtered at the glomerulus has already been re-absorbed by the time the filtrate reaches this segment of the kidney. Any remaining monohydrogen phosphate (HPO_4_^2−^) that has not yet buffered a proton (H^+^) can bind to a proton secreted by the H^+^-ATPase or the H^+^/K^+^ATPase. Ammonia from the medulla is transported to the luminal fluid by the Rhesus glycoproteins (Rhcg and Rhbg) and binds to protons, forming ammonium (NH_4_^+^). Protons and bicarbonate are produced via the actions of intracellular carbonic anhydrase II (CA II). Bicarbonate is reabsorbed via the basolateral chloride–bicarbonate exchanger. Chloride exits via a chloride channel for reuse by this exchanger.

On average, the daily non-volatile, or fixed, acid load for an individual on a Western diet is approximately 1 milliequivalent per kilogram of bodyweight. The kidneys are responsible for excreting these non-volatile acids and replenishing consumed bicarbonate to maintain normal systemic bicarbonate concentration and pH. The principal means by which the kidneys achieve these goals is through the catabolism of glutamine in the proximal tubules, which forms two ammonium ions (NH_4_^+^) and two bicarbonate ions (Figure 1). Ammonium is excreted, and bicarbonate is delivered to the systemic circulation to replenish any bicarbonate anions that were consumed through the initial acid buffering. Another major means by which the kidneys regulate acid balance is through secretion of protons, which can bind to either ammonia (NH_3_) or a titratable buffer, such as monohydrogen phosphate (HPO_4_^2−^) (Figure 1). If protons are ultimately excreted through these “proton carriers”, any bicarbonate generated in the proximal tubule or intercalated cells (Figure 2) is also delivered to the systemic circulation [18].

In CKD, metabolic acidosis develops because the daily acid load exceeds the ability of the kidneys to excrete protons and generate bicarbonate ions [19,20]. The typical Western diet is generally acid forming because of a low consumption of base-producing fruits and vegetables in relation to acid-forming animal-based protein and sodium chloride. Acid excretion is impaired because of the loss of functioning nephrons and tubule cell dysfunction from tubulointerstitial fibrosis. This tips the balance toward an acid-retaining state in CKD and, over time, metabolic acidosis. Certain medications used in patients with CKD may also contribute to the development of metabolic acidosis. These include angiotensin-converting enzyme inhibitors (ACE-is), angiotensin receptor blockers (ARBs), and sodium–glucose co-transporter-2 inhibitors (SGLT2-is). ACE-is and ARBs can lower serum bicarbonate by reducing aldosterone-mediated H^+^ secretion in the collecting duct, resulting in acid retention [16]. In addition to inhibiting sodium–glucose reabsorption in the proximal tubule, SGLT2-is also block the sodium–hydrogen exchanger antiporter (NHE3) and, therefore, proximal H^+^ secretion [21]. In severe cases, SGLT2-is can cause life-threatening ketoacidosis with normal or mildly increased serum glucose levels [22]. On the other hand, thiazide and loop diuretics may raise the serum bicarbonate concentration [16]. However, diuretics should not be prescribed solely for the purpose of raising the serum bicarbonate concentration.

Metabolic acidosis is manifested clinically by a reduction in the bicarbonate concentration, commonly measured as the total CO_2_ in a venous sample. Although arterial samples are considered as being the gold standard method for evaluating the systemic acid–base balance, arterial puncture is more difficult than venipuncture, painful, and carries some risk of causing hand ischemia if the collateral circulation is impaired. The diagnosis of chronic metabolic acidosis in CKD is typically made when the venous total CO_2_ is below 22 mEq/L in more than one measurement [23,24,25]. At this point, treating the chronic metabolic acidosis of CKD is generally recommended, with the hope that it will improve patient outcomes.

However, there is good evidence that positive acid balance is present in patients with CKD who do not have a reduced bicarbonate or total CO_2_ concentration. Vallet et al. showed that the urinary net acid excretion (NAE) was lower than the net endogenous acid production (NEAP) in patients with CKD, yet the mean serum total CO_2_ concentration was in the normal range [19]. Urinary ammonium is a major determinant of kidney acid excretion, and its urinary excretion declines as CKD progresses. In a *post hoc* study of African Americans with CKD attributed to hypertension, lower urinary ammonium levels were associated with a 2.5-fold increased risk of incident metabolic acidosis in one year [26]. Both studies also showed that lower urinary ammonium levels were associated with a higher risk of CKD progression, independent of the serum total CO_2_ concentration [19,26]. These observations demonstrate that (i) acid retention occurs in patients with CKD who have an apparently normal acid–base balance, a state often referred to as subclinical metabolic acidosis or eubicarbonatemic metabolic acidosis; (ii) urinary ammonium measurements may identify individuals with CKD who might benefit from alkali before overt metabolic acidosis develops; (iii) bone buffering may offset the imbalance between the acid load and kidney acid excretion to maintain normal pH and bicarbonate concentration [4]; and (iv) treating subclinical metabolic acidosis in CKD, when the serum bicarbonate or total CO_2_ is normal, may have a positive effect on bones, kidneys, and potentially other organs in patients with CKD.

## 3. Adverse Effects of Chronic Metabolic Acidosis in CKD

The kidneys are both the cause of chronic metabolic acidosis in CKD and the recipient of acid-mediated organ damage. Known mechanisms that contribute to acid-mediated kidney injury are actually compensatory responses that are invoked to prevent metabolic acidosis. These include enhanced kidney ammonium production and renin–angiotensin–aldosterone system (RAAS) and endothelin-1 (ET-1) activities to facilitate acid excretion by the kidneys. Increased tissue levels of ammonium promote tubulointerstitial fibrosis via the activation of the alternative pathway of complement within the kidneys [27,28,29,30]. ET-1 stimulates proximal and distal Na^+^/H^+^ exchanges, lowers distal bicarbonate secretion, and raises H^+^-ATPase activity by promoting adrenal aldosterone secretion [31,32]. Detrimentally, ET-1 is proinflammatory and stimulates kidney fibrosis through transforming growth factor-β1 (TGF-β1) and connective tissue growth factor [32,33,34,35,36,37,38]. Angiotensin II (AT-II) is also implicated in acid-mediated kidney damage. Its activity is increased in response to metabolic acidosis to facilitate proximal Na^+^/H^+^ activity and ammonia exit to the tubular fluid compartment. AT-II also stimulates aldosterone and ET-1, which can increase H^+^ secretion through increasing H^+^-ATPase activity in the intercalated cells of the distal nephron [39,40]. Although these adaptive responses to mitigate the acid burden have detrimental effects on kidney health, acid accumulation may also contribute to kidney damage through other pathways, including inflammation, insulin resistance, and oxidative stress [41,42,43,44,45]. The adverse effects of acid accumulation on kidney health are supported by results from observational studies in individuals with CKD, including children and kidney transplant recipients. In these studies, low serum total CO_2_ was identified as an independent risk factor for worsening kidney function and death [8,9,10,11,12,13,14,15]. Low total CO_2_ has also been linked with incident CKD, suggesting a potential link between dietary acid intake and the development of CKD [46,47,48].

Metabolic acidosis is also known to be associated with bone demineralization and muscle catabolism [4,5,6,7]. Pioneering studies have found that acidification increases the activity of osteoclasts, inhibits the activity of osteoblasts, and causes hypercalciuria and negative calcium balance in humans [5,49,50]. Lower serum bicarbonate concentrations have also been associated with lower bone mineral densities in humans [51] and a higher rate of loss of the bone mineral content [52]. An in-depth evaluation of bone quality in CKD patients with metabolic acidosis showed that it was associated with increased levels of bone turnover markers and lower bone density at the radius. Longitudinal changes showed cortical expansion of the bone mineral; however, there was evidence of deterioration in trabecular bone [53]. With respect to protein and muscle, metabolic acidosis causes protein catabolism by the acidification-dependent activation of ubiquitin protein ligases [6]. Results from observational studies in humans support a functional impact on muscle performance, as lower serum bicarbonate concentrations are associated with lower strength and poorer cardiorespiratory fitness and physical function [54,55,56]. These findings are a major reason that the correction of metabolic acidosis with alkali therapy is recommended.

Results from observational studies also demonstrate that metabolic acidosis is associated with an increased risk of all-cause mortality [9,57,58]. The cause of death is likely related to an increased risk of cardiovascular events, which have been described in the Systolic Blood Pressure Intervention Trial (SPRINT), kidney transplant recipients, and a large outpatient cohort [2,3,59]. Mechanisms linking metabolic acidosis with adverse cardiovascular outcomes are likely to be multifactorial but potentially include inflammation, oxidative stress, and vascular calcification.

## 4. Effects of Pharmacological Alkali Therapy in CKD

**Figure 3:**
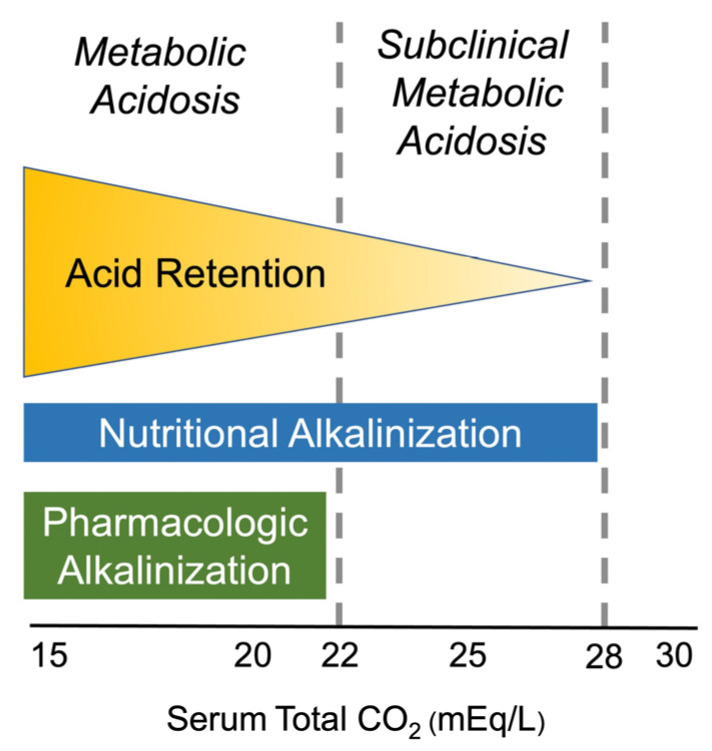
Conceptual framework of the spectrum of acid retention in chronic kidney disease. Patients with a serum total CO_2_ < 22 mEq/L have metabolic acidosis and the highest level of acid retention. More mild degrees of acid retention (subclinical metabolic acidosis) are likely present when the serum total CO_2_ is 22–28 mEq/L. Above a serum total CO_2_ of 28 mEq/L, the acid–base disorder could either be metabolic alkalosis or respiratory acidosis with metabolic compensation. For the purpose of alkalinization, nutritional therapies can be considered if the serum total CO_2_ is ≤28 mEq/L so long as the serum potassium concentration is monitored. Generally, the serum potassium should be maintained <5.0 mEq/L. Pharmacological interventions should be reserved for those with total CO_2_ < 22 mEq/L.

The pharmacological treatment of metabolic acidosis in CKD is straightforward, and therapy is generally started when the serum total CO_2_ is consistently less than 22 mEq/L (Figure 3). Sodium-based alkali salts are generally preferred to potassium-based salts, given the risk of developing hyperkalemia in patients with CKD, who are often also treated with RAAS inhibitors (RAASis). It is worth mentioning that the provision of alkali with potassium salts may be advantageous because of concerns about the sodium load on the blood pressure and urinary calcium excretion. A meta-analysis of trials in which sodium bicarbonate was the intervention did not find that sodium bicarbonate increased blood pressure [60]. In terms of urinary calcium, sodium bicarbonate was not found to increase its excretion in a secondary analysis of the Bicarbonate Administration to Stabilize Estimated Glomerular Filtration Rate (BASE) Pilot Trial [61]. Potassium-based alkalis could be considered in select patients so long as potassium is monitored closely. Potassium bicarbonate is not readily available. On the other hand, potassium citrate is widely available because it is commonly used to treat patients with nephrolithiasis.

In terms of sodium-based alkalis, sodium bicarbonate is used more often than sodium citrate and has received the most attention in recent clinical trials, apart from the investigational gastrointestinal hydrochloric acid binder veverimer [62]. Sodium bicarbonate has been used for decades and is generally safe and well-tolerated. Hence, there has been significant enthusiasm that this inexpensive intervention might mitigate the adverse effects of metabolic acidosis.

### 4.1. Kidney Effects

Results from a few studies with small-to-moderate sample sizes and at least two years of follow-up support the hypothesis that treating overt metabolic acidosis with sodium bicarbonate preserves kidney function (Table 1) [37,63]. The Use of Bicarbonate in CKD (UBI) Study is the largest sodium bicarbonate trial conducted thus far (*N* = 796), and active treatment reduced the risks of doubling of the serum creatinine by 64%, end-stage kidney disease (ESKD) by 50%, and death by 57% [64]. Pharmacological alkali therapy may also preserve kidney health in individuals with CKD who have normal serum bicarbonate concentrations, i.e., those with subclinical metabolic acidosis [65].

It is important to note that other studies have not demonstrated a positive effect of alkali therapy in patients with kidney disease and metabolic acidosis. These include a two-year trial conducted in kidney transplant recipients, called the Preserve-Transplant Study [66], and the BiCARB Trial, in which sodium bicarbonate treatment was associated with higher rates of hospitalization and worse physical performance [67]. The VALOR–CKD trial, which tested the effect of the gastrointestinal binder veverimer on CKD progression in patients with overt metabolic acidosis, also did not demonstrate a positive effect on kidney health [62]. Other studies involving individuals with CKD and subclinical metabolic acidosis (i.e., normal serum total CO_2_ concentrations) also did not demonstrate a positive effect of sodium bicarbonate on kidney health, including the VA–Bicarb Study [68] and the Alkali Therapy in CKD Trial [69]. The BASE Pilot Trial also observed a dose-dependent increase in the urinary albumin-to-creatinine ratio (ACR), which might suggest potential harm with this therapy [70]. This finding may have been spurious because it has not been observed in other trials. Future sodium bicarbonate trials should certainly monitor changes in the ACR. Thus, the effects of alkali therapy on kidney health in individuals with CKD are inconclusive. Although the results of more recent trials, such as the Preserve-Transplant Study and the VALOR–CKD Trial, may have been negative, there may be sufficient equipoise to conduct a well-powered clinical trial to determine if sodium bicarbonate treatment has positive kidney effects in patients with CKD.

**Table 1:** Summary of published pharmacological intervention trials evaluating effects on kidney function and injury.

Study	Population	Baseline Bicarbonate (mEq/L)	Total Sample Size	Intervention	Duration	Main Findings
UBI [64]	CKD stages 3–5	18–24	795	NaHCO_3_ to target bicarbonate 24–28 mEq/L	36 months	Lower risks of serum creatinine doubling, renal replacement therapy, and death
BiCARB [67]	Age > 60 eGFR < 30	<22	300	NaHCO_3_ up to 3000 mg/d	24 months	No significant effect on short physical performance battery after 12 months. Shorter 6-minute walking distance and reduction in handgrip strength in treatment group. More adverse events with treatment
Alkali Therapy in CKD [69]	CKD stage 3 or 4	20–26	149	NaHCO_3_ 0.4 mEq/kg (ideal bodyweight/day)	24 months	No significant effects on bone mineral density, sit-to-stand time, other physical function assessments, and eGFR
BASE Pilot Trial [70]	CKD stage 3b or 4; or CKD stage 3a with ACR ≥ 50 mg/g	20–28	192	NaHCO_3_ 0.5 or 0.8 mEq/kg (lean bodyweight/day)	28 weeks	No significant effects on blood pressure and weight Dose-dependent increase in sodium bicarbonate levels Dose-dependent increase in urinary ACR
VA–Bicarb Trial [68]	Diabetic CKD stages 2–4 with ACR > 30 mg/g	22–28	74	NaHCO_3_ 0.5 mEq/kg lean bodyweight/day	6 months	No statistically significant effects on urinary markers of kidney injury
Veverimer (40-week extension study) [71]	eGFR 20–40 mL/min per 1.73 m^2^	12–20	196	Veverimer 6g/day then titrated to target bicarbonate level (22–29 mEq/L)	52 weeks	3% in veverimer vs. 10% in placebo; discontinued treatment Treatment with veverimer improved physical function. Fewer treated with veverimer died or progressed to ESKD.
VALOR–CKD Trial [62]	eGFR 20–40 mL/min per 1.73 m^2^	12–20	1480	Veverimer vs. placebo	Mean follow-up: 26.7 months	Higher-than-expected bicarbonate level in placebo group No statistically significant difference in kidney events
De brito-Ashurst et al. [63]	CKD stages 4–5	17–19	134	NaHCO_3_ titrate to ≥23 mEq/L vs. usual care	2 years	Lower CrCl decline (−1.88 vs. −5.93 mL/min) Lower relative risk of ESKD 0.13 (95% CI, 0.04–0.40)
Phisitkul et al. [37]	eGFR 20–60, Hypertension	<22	59	Sodium Citrate 1 mEq/kg/d vs. usual care	2 years	Lower eGFR decline (−3.6 vs. −8.7 mL/min/1.73 m^2^)
Mahajan et al. [65]	CKD stage 2, Hypertension, ACR ≥ 300	-	120	NaHCO_3_ 0.5 mEq/kg/d vs. equimolar NaCl vs. placebo	5 years	Lower eGFR decline (−6.8 vs. −10.8 vs. −12.7 mL/min/1.73 m^2^)
Preserve-Transplant Study [66]	Kidney transplant recipient >1 year, eGFR 15–89	<22	242	NaHCO_3_ 1.5–4.5 g/d vs. placebo	2 years	No difference in eGFR between the groups.

### 4.2. Bone Effects

Studies on the effects of alkali supplementation on bone health in CKD are limited. In one study, treatment of metabolic acidosis with sodium bicarbonate mildly attenuated increases in intact parathyroid hormone (iPTH) levels over three months [72]. In another study of individuals with CKD and overt metabolic acidosis, sodium bicarbonate did not have significant effects on iPTH, C-terminal telopeptide of type I collagen (CTX-1), or procollagen type I intact N-terminal propeptide (P1NP). However, sodium bicarbonate increased serum intact fibroblast growth factor-23 (iFGF-23) levels, which have been associated with a higher risk of cardiovascular complications [73]. A secondary analysis of the BASE Pilot Trial also did not observe any effects of treatment with sodium bicarbonate on iPTH, CTX-1, PINP, iFGF-23, bone-specific alkaline phosphatase (B-SAP), tartrate-resistant acid phosphatase 5b (TRAP5b), or vitamin D metabolites [61]. The Alkali Therapy in CKD Trial also did not find a positive effect of sodium bicarbonate on bone mineral density (BMD) relative to a placebo [69]. However, in kidney transplant recipients with metabolic acidosis, potassium citrate improved bone histomorphometry and preserved B-SAP, CTX-1, and P1NP levels but had no effect on iPTH, vitamin D metabolite levels, or BMD over 1 year [74]. The strongest evidence supporting a beneficial effect of alkali therapy on bone comes from studies in post-menopausal women who did not have CKD, and most of them were treated with potassium-based alkalis [75,76,77]. Whether this is because of differences in the cation (potassium vs. sodium) or simply because bone health is impacted by a variety of factors in patients with CKD is uncertain. A major gap, as it relates to the correction of metabolic acidosis, is that surrogate measures of bone health have been used to characterize the effects of this information. Fracture outcomes would be quite informative.

### 4.3. Effects on Muscle and Protein

The effects of treating metabolic acidosis on skeletal muscle health and/or protein catabolism have not been studied nearly as well as the effects on kidney or bone health in CKD.

Perhaps the best evidence comes from a randomized, open-label study in which the correction of metabolic acidosis with sodium bicarbonate (*N* = 67) increased the mid-arm muscle circumference, lowered the protein nitrogen appearance, and increased serum albumin over two years relative to the placebo group (*N* = 67) [63]. In a single-arm study of 20 CKD patients with serum total CO_2_ levels of 20–24 mEq/L, sodium bicarbonate improved sit–stand times and reduced urine urea nitrogen excretion, suggesting that protein catabolism was diminished with treatment. However, there was no effect on handgrip strength, suggesting that alkalis might have greater impacts on lower-extremity muscles [78].

### 4.4. Cardiovascular Effects

The cardiovascular effects of treating metabolic acidosis in CKD are unclear. On the one hand, there are two major cardiovascular concerns with sodium-based alkalis. First, the sodium load may cause fluid retention and increase blood pressure. In a meta-analysis of five trials that administered either sodium bicarbonate or sodium citrate to participants with CKD, there was no significant increase in the total bodyweight, systolic blood pressure, or diastolic blood pressure. However, treatment was associated with 38% higher relative risk of escalating antihypertensive therapy and 39% higher relative risk of escalating diuretic therapy [79]. A subsequent meta-analysis of 14 trials that included over 2000 individuals did not find that sodium bicarbonate increased systolic blood pressure or led to an increase in antihypertensive or diuretic therapy [60]. In experimental animal models of hypertension (Dahl salt-sensitive rats, chronic-angiotensin-II-infused rats, and deoxycorticosterone acetate-salt-fed rats), blood pressure was found to increase only if sodium was accompanied by the chloride anion but not other anions, including bicarbonate, suggesting that the effects of sodium on blood pressure are present only when chloride is the co-administered anion [80,81,82,83,84,85].

A second cardiovascular concern is that sodium bicarbonate may promote vascular calcification. Vascular calcification is common in patients with CKD and is associated with all-cause and cardiovascular mortalities [86]. Evidence supporting this concern largely comes from animal studies in which calcification was enhanced in alkalotic environments and diminished in acidotic environments [87,88,89]. However, there has not been any evidence in humans that sodium bicarbonate worsens vascular calcification.

On the other hand, metabolic acidosis has been linked with increased risks of death and cardiovascular events, and the results from the UBI Study suggest that the treatment of metabolic acidosis in CKD reduces mortality [64]. One potential mechanism is by improving vascular function, as measured by flow-mediated dilation (FMD) [73]. This mechanism of cardioprotection may be unique to patients with overt metabolic acidosis, as treatment with sodium bicarbonate in individuals with CKD and normal bicarbonate concentrations did not affect FMD [90]. Another potential mechanism may be through increasing serum klotho levels, and higher serum klotho levels are associated with a lower risk of cardiovascular disease [91]. In a *post hoc* analysis of the BASE Trial, sodium bicarbonate was found to have a dose-dependent increase in serum klotho levels over 28 weeks [61]. The mechanism by which sodium bicarbonate increases serum klotho levels is likely because of an increase in urinary pH. The increase in pH activates the calcium-sensing receptor (CaSR) in the distal convoluted tubule, which abundantly expresses klotho on the cell surface. The stimulation of CaSR activates the protease ADAM10 (A disintegrin and metalloproteinase 10), which cleaves membrane-bound klotho for delivery to the systemic circulation [92]. As klotho is considered as being an “anti-aging” protein, future studies investigating the cardiovascular effects of bicarbonate therapy on klotho and related pathways should be considered.

## 5. Nutritional Alkali Therapy in CKD

Pharmacological therapies provide base as either bicarbonate or citrate, the latter is metabolized to bicarbonate in the liver, to mitigate acid excess in CKD. The Western diet is highly acid-forming owing to a low intake of base-producing fruits and vegetables relative to animal protein, preservatives, and sodium chloride. Animal protein is high in methionine and cysteine, which are sulfur-containing amino acids, that are net acid-forming. Hence, nutritional counseling can be an effective strategy to mitigate acid excess in CKD. Simply stated, increasing fruits and vegetables in the diet while reducing the consumption of animal protein can be recommended for motivated patients so long as serum potassium is monitored.

Evidence to support these recommendations comes from observational and interventional trials. In observational studies, higher dietary acid loads have been associated with lower eGFR in National Health and Nutrition Estimation Survey (NHANES) 1999–2004 participants [93]. Further, NHANES III participants with CKD who were in the highest tertile of the dietary acid load had a 3-fold increased risk of progressing to end-stage kidney disease (ESKD) [94]. Kidney transplant recipients with higher dietary acid loads are also at risk of graft loss [95]. The Dietary Approaches to Stop Hypertension (DASH) diet can be a useful tool to counsel patients on how to consume a base-producing diet. In studies examining adherence to a DASH diet, poorer adherence was associated with both the development of CKD and progression from established CKD to ESKD [96,97].

Results from interventional studies, primarily conducted in patients with CKD attributed to hypertension, back the findings from these observational studies [98,99,100]. In general, participants in these studies were prescribed a diet rich in fruits and vegetables to lower PRAL by 50%. Among those with stage 2 CKD and serum bicarbonate levels >24.5 mEq/L, fruits and vegetables decreased urinary markers of kidney damage, including ACR, TGF-β1, and N-acetyl beta-D-glucosaminidase (NAG), over 30 days [98]. For individuals with stage 3 CKD and serum bicarbonate levels of 22–24 mEq/L, treatment with fruits and vegetables to reduce PRAL by 50% or with sodium bicarbonate (0.3 mEq/kg/d) preserved eGFR similar to each other and better than usual care over 36 months [100]. Notably, both of these trials were conducted in individuals who did not have metabolic acidosis. This is particularly important because only 15% of CKD patients have metabolic acidosis, suggesting a strong role for nutritional interventions in early-stage CKD patients. The benefits of nutritional alkali therapy seem to also extend to individuals with more advanced stage 4 or 5 CKD with overt metabolic acidosis as well. In a study of individuals with stage 4 CKD and serum bicarbonate levels <22 mEq/L, the fruit-and-vegetable diet also lowered urinary ACR, TGF-β1, and NAG [99]. The impacts of fruits and vegetables on kidney events should be determined in patients with other forms of CKD, in particular, diabetes mellitus, because these studies were conducted in individuals with CKD attributed to hypertension, who generally have a lower risk of CKD progression.

It is important to mention that there are other ways in which diets containing fruits and vegetables may be kidney protective apart from mitigating the dietary acid load. Fruits and vegetables are low in salt content, which could help to control blood pressure. They are also low in bioavailable phosphorus, which could mitigate vascular calcification and left ventricular hypertension through its link with FGF-23. Lastly, the accompanying dietary fiber could increase the gastrointestinal transit time, positively alter the gut microbiome, and reduce the production and absorption of uremic toxins.

Another strategy to mitigate the net acid load through nutrition is by lowering the consumption of animal protein. Although many protein-restriction studies have been conducted, the most widely known is the Modification of Diet in Renal Disease (MDRD). The low-protein diet (0.58 g/kg/d) prescribed in MDRD did not clearly demonstrate a benefit on kidney function compared to the usual protein diet (1.3 g/kg/d) [101]. The very low protein diet (0.28 g/kg/d) was associated with higher mortality [102], and, therefore, excessive protein restriction is not recommended.

Nutritional strategies for the purpose of alkalinization should be considered for all patients with CKD who have a serum total CO_2_ ≤ 28 mEq/L (Figure 1) because values above this would be consistent with either metabolic acidosis or respiratory acidosis with metabolic compensation. The main risk with consuming a diet supplemented with fruits and vegetables is hyperkalemia. Serum potassium should be closely monitored in patients who choose to implement a base-producing diet. In studies by Goraya and colleagues, the average dose of fruits and vegetables added to the usual diet was 2–4 cups per day [103]. Mitigating animal protein intake can counter the acid load by reducing cysteine and methionine intake, which are oxidized to sulfuric acid. Reducing animal protein and/or incorporating plant-based protein can be considered. Kidney nutrition specialists are an excellent resource for patients and family members.

## 6. Summary

Metabolic acidosis is a frequent complication of CKD and is associated with a number of adverse outcomes, including CKD progression, poor musculoskeletal health, cardiovascular events, and death. Although there is general enthusiasm that treating metabolic acidosis improves patient outcomes, the existing evidence is conflicting. The largest sodium bicarbonate trial, the UBI Study, demonstrated a lower risk of CKD progression, ESKD, and mortality [64]. However, in kidney transplant recipients, a positive effect on kidney outcomes was not observed, potentially because the transplanted kidney is subjected to additional mechanisms of kidney damage that cannot be overcome by alkali therapy alone [66]. In addition, the treatment of metabolic acidosis with the gastrointestinal hydrochloric acid binder veverimer did not have a positive effect on kidney events [62]. There has also been enthusiasm to study the effects of sodium bicarbonate treatment in patients with normal serum bicarbonate concentrations. Although the results from some of these trials support the hypothesis that treating subclinical metabolic acidosis preserves kidney function, other trials have been unable to replicate those findings. The negative results of more recent trials may have re-established the clinical equipoise needed to conduct well-powered clinical trials to determine the efficacy of alkali therapy in patients with CKD. Mitigating the dietary acid load by adding fruits and vegetables to the diet and reducing the consumption of animal-based protein, preservatives, and sodium chloride are other strategies to counter the effects of acid on the kidneys and other organs. Such diets have other positive health effects, but close attention to the serum potassium concentration is warranted in patients with CKD.

## Data Availability

Not applicable.
